# Mobile group I introns at nuclear rDNA position L2066 harbor sense and antisense homing endonuclease genes intervened by spliceosomal introns

**DOI:** 10.1186/s13100-022-00280-4

**Published:** 2022-10-08

**Authors:** Kjersti Lian, Betty M. N. Furulund, Anders A. Tveita, Peik Haugen, Steinar D. Johansen

**Affiliations:** 1 Nofima AS, Muninbakken 9-13, Breivika, 9291 Tromsø, Norway; 2 Genomics division, Faculty of Biosciences and Aquaculture, Nord University, N-8049 Bodø, Norway; 3 Medical Department, Bærum Hospital, Vestre Viken Hospital Trulst, Drammen, Norway; 4 Department of Chemistry and Center for Bioinformatics, Faculty of Science and Technology, UiT-The Arctic University of Norway, N-9037 Tromsø, Norway

**Keywords:** Antisense, *Diderma*, *Didymium*, Homing endonuclease, Intron evolution, Mobile DNA, Myxomycete, Ribozyme

## Abstract

**Background:**

Mobile group I introns encode homing endonucleases that confer intron mobility initiated by a double-strand break in the intron-lacking allele at the site of insertion. Nuclear ribosomal DNA of some fungi and protists contain mobile group I introns harboring His-Cys homing endonuclease genes (HEGs). An intriguing question is how protein-coding genes embedded in nuclear ribosomal DNA become expressed. To address this gap of knowledge we analyzed nuclear L2066 group I introns from myxomycetes and ascomycetes.

**Results:**

A total of 34 introns were investigated, including two identified mobile-type introns in myxomycetes with HEGs oriented in sense or antisense directions. Intriguingly, both HEGs are interrupted by spliceosomal introns. The intron in *Didymium squamulosum*, which harbors an antisense oriented HEG, was investigated in more detail. The group I intron RNA self-splices in vitro, thus generating ligated exons and full-length intron circles. The intron HEG is expressed in vivo in *Didymium* cells, which involves removal of a 47-nt spliceosomal intron (I-47) and 3′ polyadenylation of the mRNA. The *D. squamulosum* HEG (lacking the I-47 intron) was over-expressed in *E. coli*, and the corresponding protein was purified and shown to confer endonuclease activity. The homing endonuclease was shown to cleave an intron-lacking DNA and to produce a pentanucleotide 3′ overhang at the intron insertion site.

**Conclusions:**

The L2066 family of nuclear group I introns all belong to the group IE subclass. The *D. squamulosum* L2066 intron contains major hallmarks of a true mobile group I intron by encoding a His-Cys homing endonuclease that generates a double-strand break at the DNA insertion site. We propose a potential model to explain how an antisense HEG becomes expressed from a nuclear ribosomal DNA locus.

**Supplementary Information:**

The online version contains supplementary material available at 10.1186/s13100-022-00280-4.

## Background

Nuclear group I introns are intervening sequences so far exclusively reported in ribosomal DNA (rDNA) that interrupt highly conserved sites in the small subunit (SSU) and large subunit (LSU) rRNA genes [1, 2]. Group I introns appear restricted to some eukaryotic linages, and among these are the ascomycete fungi and various groups of protists such as the ciliates, amoebo-flagellates, green algae and myxomycetes. About 100 rDNA intron insertion sites are currently known, equally distributed between the SSU and LSU rRNA genes [3–5]. A common nomenclature of rDNA group I introns, based on the *E. coli* rRNA gene numbering system, has been established [6].

Group I introns encode ribozymes responsible for RNA processing reactions, and occasionally contain endonuclease genes involved in intron homing mobility. A group I ribozyme possesses a characteristic structural fold at the secondary and tertiary levels [7], organized into three structural domains (catalytic, substrate and scaffold) and at least nine paired segments (P1 to P9) [8]. Two subgroups (group IC1 and group IE) have been noted among the nuclear group I introns [2]. The main structural difference between the subgroups is found in the scaffold domain where group IC1 introns have a larger and more complexed P5 RNA structure than group IE introns. Detailed structural information is available from the *Tetrahymena* group IC1 intron (Tth.L1925) ribozyme based on RNA crystallization and cryo-EM [9–11], and a computer-based model reports three-dimensional structural features of the *Didymium* group IE intron (Dir.S956–1) ribozyme [12].

Some nuclear group I introns are capable of self-splicing in vitro as naked RNA following a two-step transesterification reaction [2, 13]. The self-splicing reaction, which is catalysed by the group I ribozyme and dependent on an exogenous guanosine cofactor (exoG), leads to perfectly ligated exon sequences and excised linear intron RNA. In an alternative and competing reaction the intron RNA may self-process independently of exoG, leading to full-length intron circles and fragmented RNA exons [2, 14].

About 5–10% of all known nuclear group I introns carry homing endonuclease genes (HEGs), and thus appear as potential mobile genetic elements [2]. Experimental support for homing mobility has been reported in two nuclear group I intron systems, Ppo.L1925 in *Physarum polycephalum* [15] and Dir.S956–1 in *Didymium iridis* [16]. Here, intron homing occurs in sexual crosses between intron-containing and intron-lacking strains. Homing is initiated by a double-strand break close to, or at, the intron insertion site, performed by the intron encoded homing endonuclease. The event is completed by homology-dependent repair that results in a highly efficient unidirectional transfer of the intron [2, 17]. All nuclear homing endonucleases belong to the His-Cys family [17–19], which act as zinc-coordinating homodimers [20] and generate tetra- or pentanucleotide 3′-overhangs at their cleavage sites [21–24].

An intriguing question is how the endonuclease proteins become expressed in an rDNA context. Current knowledge suggests that the mRNAs are generated from processed RNA pol I transcripts (sense HEGs) or from a separate RNA pol II transcript (antisense HEGs) [25]. Some mRNAs are 5′-capped by a 2′,5′ lariat catalyzed by a separate ribozyme [26–29], and most appear polyadenylated [24, 29–33]. Furthermore, some HEGs also harbor small spliceosomal introns that need to be removed [30, 32, 33] and thus may participate in homing endonuclease expression. One intriguing and unsolved question remains. How are HEGs that are organized on the antisense strand expressed? Expression of such HEGs has the potential to cause serious problems for normal cell growth since their mRNAs may induce antisense interference to precursor rRNA transcripts. The *D. iridis* intron Dir.S956–2 was reported to carry and express an antisense HEG from a potential internal promoter, and its mRNA maturation includes both polyadenylation and spliceosomal intron removal [24, 34].

Here we characterized 34 nuclear L2066 group I introns from myxomycete and ascomycete rDNA and identified two differently organized mobile-type introns (Dal.L2066 and Dsq.L2066) in *Diderma alpinum* and *D. squamulosum* with HEGs in sense and antisense orientation, respectively. A common feature to both HEGs was consensus polyadenylation signals and the presence of spliceosomal introns. The antisense HEG intron Dsq.L2066 was investigated in more detail including in vitro self-splicing activity, in vivo HEG expression, as well as homing endonuclease activity and cleavage site mapping.

## Results

### Structural features of group I introns at position L2066 in the nuclear LSU rRNA gene

**Fig. 1:**
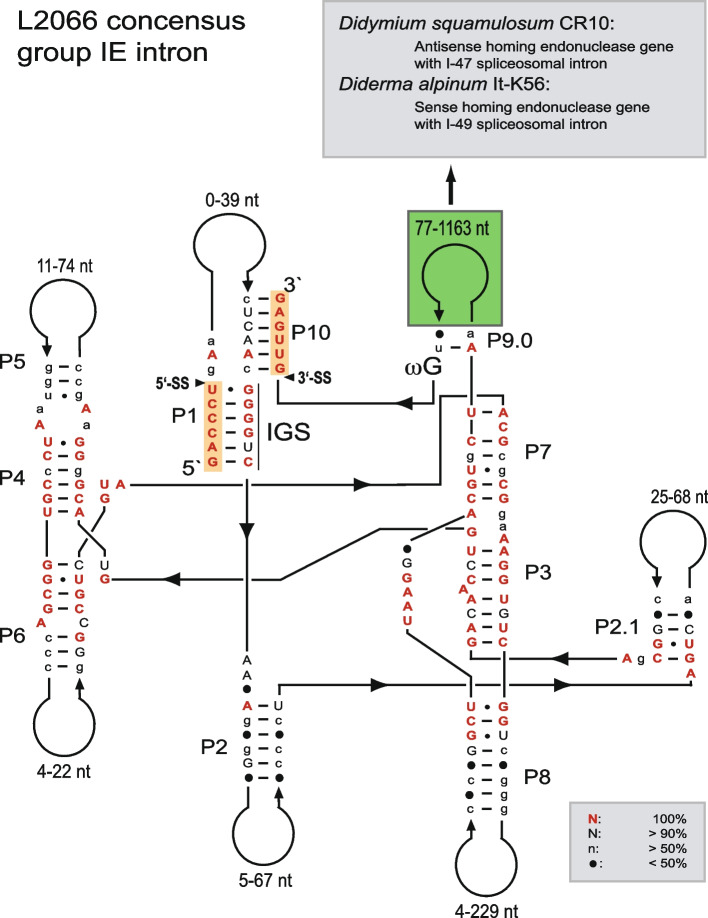
Consensus secondary structure diagram of L2066 group I introns in myxomycetes and ascomycetes based on 153 nucleotide positions in the catalytic core common among all introns from 34 taxa (see Table 1). Sequence size variations are noted in peripheral regions, and homing endonuclease genes (HEGs) are found as P9 extensions. P1-P10, paired RNA segments; 5′ SS and 3′SS, exon-intron splice sites. Invariant nucleotide positions are shown as red uppercase letters. Black uppercase letter, > 90% conservation; lowercase letters, ≥ 50% conservation; filled circles, < 50% conservation

Introns at site L2066 site in LSU rRNA (*E. coli* LSU rRNA numbering [6]) disrupt helix 74 in domain V, which is in proximity to the peptidyl transferase center. L2066 group I introns have so far been noted in two orders of myxomycete protists and three orders of ascomycete fungi (Table 1). Consensus structure diagrams of myxomycete (16 taxa) and ascomycete (18 taxa) L2066 group I intron RNA are shown in Fig. 1 and Fig. S1, which highlight conserved core structures and more variable peripheral regions. The introns fold into a typical group IE ribozyme organization including a less complex P4-P6 scaffold domain, a core-stabilizing P13 helix, and strong exon-binding segments (P1 and P10).

**Table 1:** Key features of L2066 group I introns in myxomycetes and ascomycetes

Host species	Strain/ isolate	L2066	HEG ^a^	Acc. Number
**MYXOMYCOTA**				
**Order: Physarales**				
*Craterium minutum*	It-IG38 (Italy)	536 bp	–	HE655081
*Diderma alpinum*	It-K56 (Italy)	1480 bp	+ (S; I-49)	HE655057
*Diderma alpinum*	Uk-K78 (Ukraine)	467 bp	–	HE655058
*Diderma meyerae*	It-K61 (Italy)	414 bp	–	HE655059
*Diderma niveum*	Fr-K10 (France)	438 bp	–	AM407429
*Diderma niveum*	It-K66 (Italy)	434 bp	–	HE655060
*Diderma niveum*	Uk-K79 (Ukraine)	434 bp	–	HE655061
*Diderma saundersii*	Mx-K30 (Mexico)	595 bp	–	AM407428
*Didymium squamulosum*	CR10 (Costa Rica)	1197 bp	+ (AS; I-47)	AM407427
*Fuligo septica*	IW1 (Iowa-USA)	389 bp	–	HE655082
*Lepidoderma aggregatum*	Uk-K86 (Ukraine)	449 bp	–	HE655062
*Lepidoderma alpestroides*	Fr-K17 (France)	435 bp	–	ON155995
*Lepidoderma carestianum*	Fr-K18 (France)	449 bp	–	AM407430
*Lepidoderma crustaceae*	It-K62 (Italy)	476 bp	–	HE655064
*Mucilago crustacea*	No-K94 (Norway)	468 bp	–	HE655067
**Order: Stemonitales**				
*Comatricha laxa*	EdHa (USA)	493 bp	–	ON155996
**ASCOMYCOTA**				
**Order: Capnodiales**				
*Sphaerulina quercicola*	CBS 663.94 (Netherlands)	367 bp	–	GU214496
**Order: Hypocreales**				
*Beauveria bassiana*	DAOM216540	358 bp	–	EU334679
*Beauveria bassiana*	ARSEF654	358 bp	–	KJ701419
*Beauveria bassiana*	ARSEF502	358 bp	–	KJ701420
*Beauveria bassiana*	ARSEF2991	359 bp	–	EU334676
*Beauveria bassiana*	VS5714 (Germany)	358 bp	–	MG654725
*Beauveria bassiana*	VSP11 (Germany)	358 bp	–	MG654726
*Beauveria bassiana*	STB	358 bp	–	JF429894
*Cordyceps kanzashiana*	(Japan)	380 bp	–	AB044639
*Cordyceps militaris*	ATCC 34164	360 bp	–	CP023322
*Cordyceps profilica*	97,009 (Japan)	379 bp	–	AB044641
*Fusarium* sp.	Uk-K90 (Ukraine)	287 bp	–	ON155007
*Levanicillium* sp.	CEP 419 (Argentina)	364 bp	–	MH013330
*Ophiocordyceps sinensis*	AMI1080 (China)	341 bp	–	FJ461354
*Ophiocordyceps sinensis*	AMI1081 (China)	341 bp	–	FJ461355
*Paecilomyces tenuipes*	(Japan)	400 bp	–	AB044642
**Order: Saccharomycetales**				
*Myxozyma monticola*	NRRL Y-17726	376 bp	–	DQ518989
*Myxozyma nipponensis*	NRRL Y-27625	342 bp	–	DQ518993

^a^(+) denotes presense of homing endonuclease gene (HEG). (S) denotes sense orientation and (AS) antisense orientation. (I-49) and (I-47) denote the presense of spliceosomal introns of 49 bp and 47 bp, respectively

**Fig. 2:**
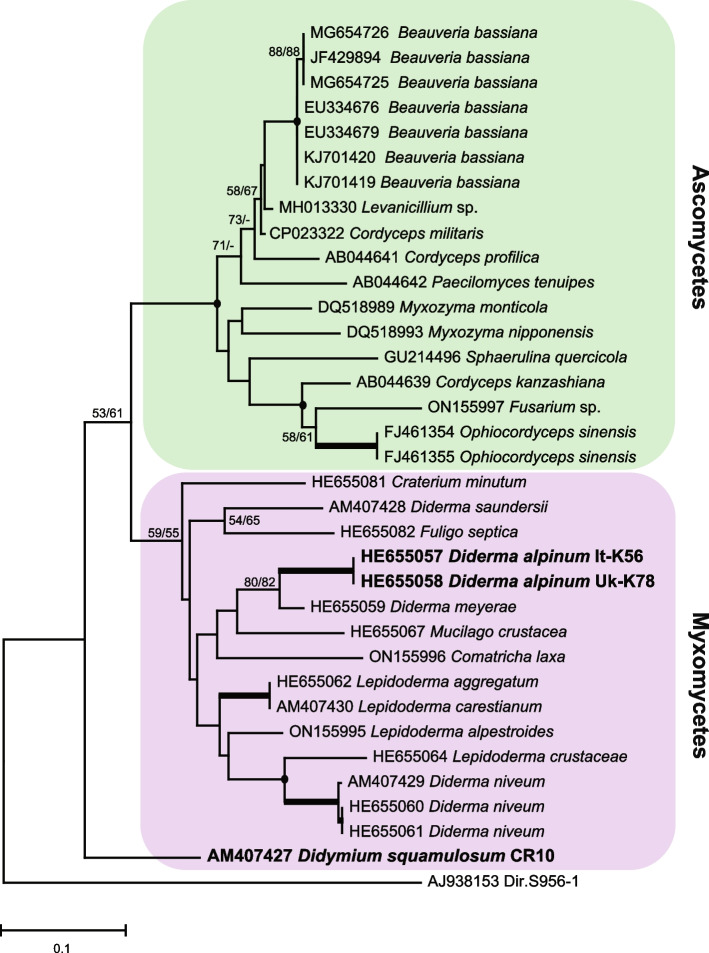
Molecular phylogeny of myxomycete and ascomycete L2066 group I introns. The intron topology was obtained by Neighbor-joining (NJ) analysis of 34 taxa and 186 nt aligned positions. The *Didymium iridis* intron Dir.S956–1 was included as an outgroup. The NJ and Minimal Evolution (ME) bootstrap replicates (≥ 50%) are given for each node (NJ/ME). Maximum support in NJ and ME (≥ 97%) are shown in bold branches. Black dots at branch points indicate NJ and ME robustness (≥ 90%). The scale-bar indicates the fraction of substitutions per site. GenBank accession numbers for taxa are shown

Structural conservation and variation among introns were further assessed by a phylogenetic analysis based on 186 aligned core sequence positions (Fig. S2) representing all the 34 taxa. A Neighbor Joining analysis is presented in Fig. 2 and shows that all ascomycete L2066 introns cluster together with high bootstrap value, but relationships among myxomycete introns appeared more scattered and not strictly organized according to current taxonomy (Table 1). Reconstructing group I intron in long-term phylogeny appears challenging due to a limited number of aligned positions, and to biological factors such as horizontal transfers and intron gain-and-loss. The L2066 introns in ascomycetes were uniform in size varying only from 287 bp to 400 bp (Table 1). Size variation was more pronounced among the myxomycete L2066 introns. All introns vary in length between 400 bp and 500 bp, except two introns that are significantly longer, i.e., 1480 bp in *D. alpinum* It-K56 and 1197 bp in *D. squamulosum* CR10 (Table 1).

### *D. alpinum* and *D. squamulosum* L2066 group I introns harbor HEGs in sense or antisense orientations that are interrupted by small spliceosomal introns

**Fig. 3:**
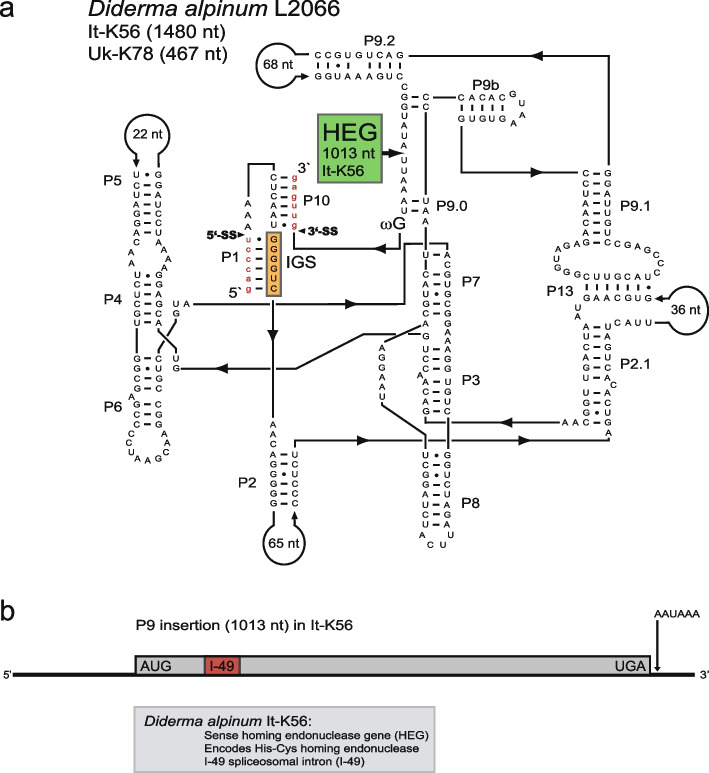
Structural organization of L2066 group I introns in *Diderma alpinum*. **a** Secondary structure diagram of the Italian (It-K56) and the Ukraine (Uk-K78) isolates. Homing endonuclease gene (HEG) insertion in It-K56 is indicated. See legend to Fig. 1a for structural annotations. **b** Schematic presentation of HEG insertion in P9 in isolate It-K56. Start and stop codons are indicated, as well as the 49 nt spliceosomal intron (I-49). The AAUAAA polyadenylation signal is located close to the termination codon

**Fig. 4:**
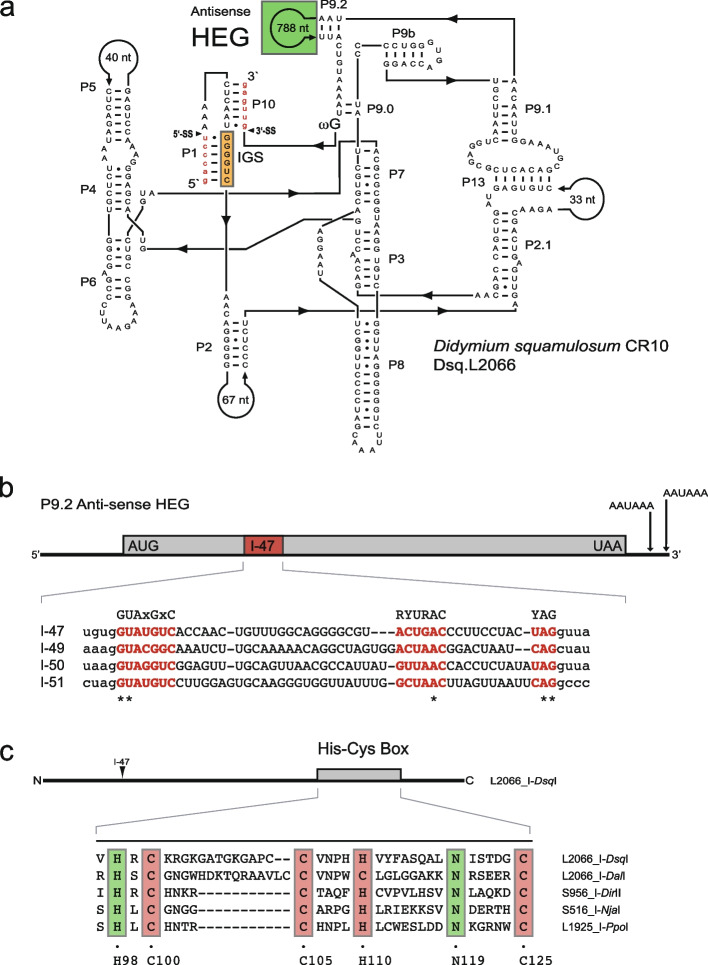
Structural organization of L2066 group I intron in *Didymium squamulosum*. **a** Secondary structure diagram of the Costa Rican (CR10) isolate. Antisense homing endonuclease gene (HEG) insertion is indicated. See legend to Fig. 1a for structural annotations. **b** Schematic presentation of HEG insertion in P9. Start and stop codons are indicated, as well as the 47 nt spliceosomal intron (I-47). Two AAUAAA polyadenylation signals are located close to the termination codon. Below is an alignment of selected spliceosomal introns in group I intron HEGs. I-47, *Didymium squamulosum* L2066; I-49, *Diderma alpinum* L2066; I-50, *Didymium iridis* S956–2; I-51, *Didymium iridis* S956–1. Yeast spliceosomal consensus sequences at branch and splice sites are indicated above the alignment. (*) indicate hallmark spliceosomal intron positions (GU-A-AG). **c** Schematic presentation of the I-*Dsq*I homing endonuclease containing a His-Cys box. Below is an amino acid alignment of His-Cys box features in *Didymium squamulosum* (I-*Dsq*I), *Diderma alpinum* (I-*Dal*I), *Didymium iridis* (I-*Dir*II), *Naegleria jamiesoni* (I-*Nja*I) and *Physarum polycephalum* (I-*Ppo*I). I-*Nja*I and I-*Ppo*I represent well-studied His-Cys homing endonucleases. Conserved residues (boxed) corresponding to those presented in the I-*Ppo*I crystal structure [20]. C100, C105, H110 and C125 are involved in zinc binding, and H98 and N119 are associated with the active site

Intron phylogeny (Fig. 2) and secondary structure diagram (Fig. 3a) show that the *D. alpinum* isolates It-K56 and Uk-K78 possess identical catalytic RNA core sequences. Interestingly, they only differ due to an insertion of 1013 nt into P9 of the Italian isolate (i.e., It-K56). A closer inspection of the P9 insertion identified a HEG, putatively encoding a homing endonuclease protein consisting of 235 amino acids (named I-*Dal*I) and belonging to the His-Cys family (see Fig. 4c). In addition, the HEG is associated with a consensus polyadenylation signal (AAUAAA). Finally, the *D. alpinum* HEG was found to be interrupted by a 49-nt spliceosomal intron (I-49) in proximal distance to the 5′ end of the reading frame (Figs. 3b and 4b).

The L2066 intron in *D. squamulosum* (named Dsq.L2066) has a more unusual structural organization (Fig. 4a). Specifically, the catalytic RNA core corresponds to a canonical group IE type structure, but the peripheral paired segment P9.2 contains a large HEG-encoding insertion. Interestingly, the HEG is positioned in an antisense orientation and is apparently transcribed from the opposite rDNA strand compared to that of the rRNA genes and intron ribozyme. Moreover, the HEG has recognizable start and stop codons, two consensus polyadenylation signals in proximity to the stop codon, and a 47-bp spliceosomal intron (I-47) closer to the 5′ end (Fig. 4b). If the I-47 is removed, then the HEG encodes for a putative homing endonuclease (named I-*Dsq*I) consisting of 204 amino acids and belonging to the His-Cys family (Fig. 4c).

### Dsq.L2066 self-splices and generates full-length intron RNA circles in vitro

**Fig. 5:**
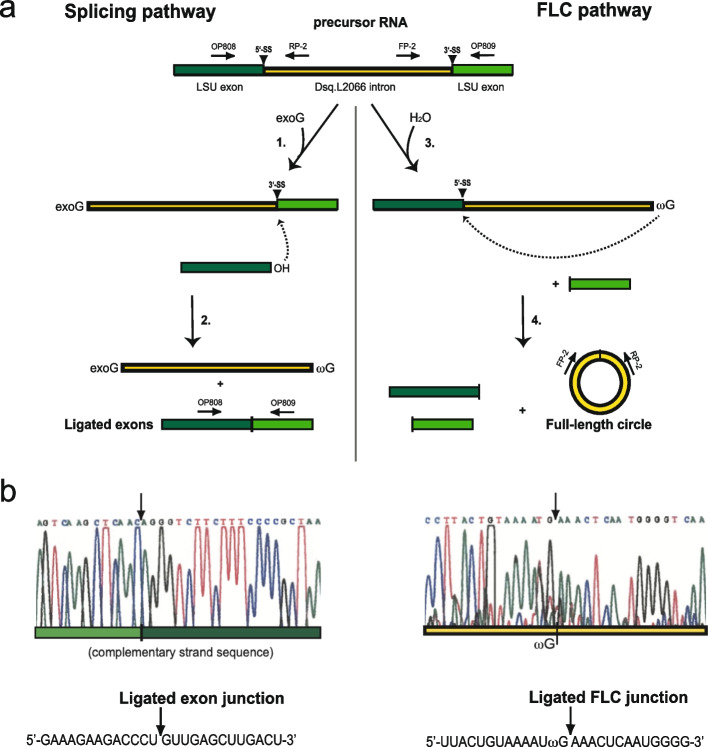
In vitro self-splicing of Dsq.L2066 RNA. **a** The two main processing pathways, splicing and full-length intron circularization (FLC), and approach of RT-PCR/ Sanger sequencing assessments. The self-splicing pathway (left) involves two transesterification reactions. The first reaction (1.) is initiated by a nucleophilic attack by an exogenous guanosine cofactor (exoG). The second reaction (2.) starts with an attack at the 3′ splice site (3′SS) and results in ligated exons and excised linear intron. The FLC pathway (right) involves hydrolysis at the 3′SS (3.) followed by a nucleophilic attack (4.) at the 5′SS by the terminal guanosine (ωG). The FLC pathway is independent of exoG and results in full-length intron circles and fragmented exons. **b** Experiments of ligated exons (left) and full-length intron circle (right) junction analyses. RNA sequences corresponding to ligated exon junction and ligated FLC junction are presented below

RNA self-splicing and processing of Dsq.L2066 was evaluated from in vitro transcribed and purified RNA, which was subsequently incubated at splicing conditions for 60 min. Ligated exons and intron circles were assessed by eluting the RNA from a polyacrylamide gel, and by subjecting the RNA to RT-PCR using specific primers (Fig. 5a). Sanger sequencing of amplified fragments confirmed self-splicing by identifying the RNA corresponding to ligated exons (Fig. 5b, left panel). Furthermore, an RNA band corresponding to full-length intron RNA circles was identified (Fig. 5b, right panel). Based on these finding we infer that Dsq.L2066 is capable of processing and self-splicing the RNA using the same pathways as previously reported for the *D. iridis* S956–1 group IE intron [14, 35, 36].

### I-*Dsq*I homing endonuclease is expressed from the rDNA antisense strand in vivo

**Fig. 6:**
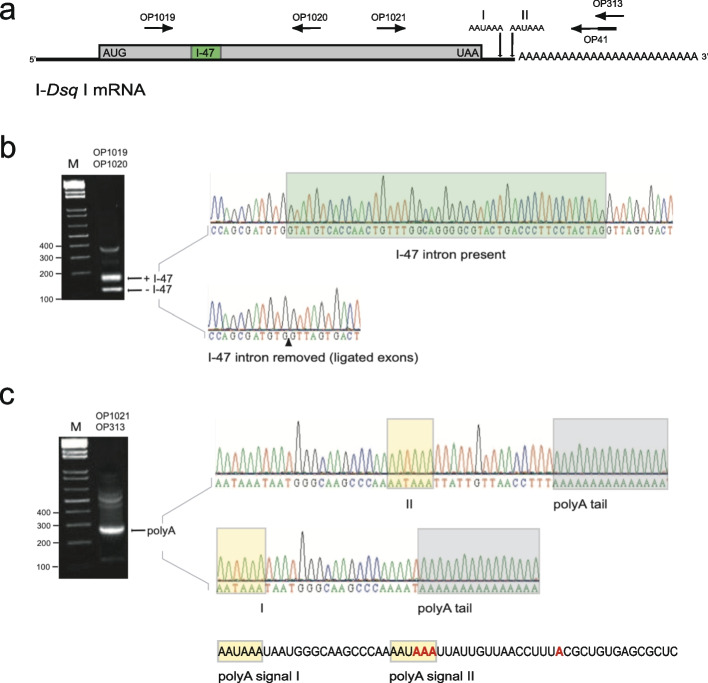
In vivo maturation of I-*Dsq*I mRNA in *Didymium squamulosum*. **a** Schematic presentation of I-*Dsq*I mRNA. Start (AUG) and stop (UAA) codons are indicated, as well as the 47 nt spliceosomal intron (I-47). Two AAUAAA polyadenylation signals (I and II) are located close to the termination codon. Primer locations used in assessment of intron removal and polyadenylation are indicated. **b** RNA from amoeba cells was analyzed by an RT-PCR approach using a primer set flanking I-47. Amplicons corresponding to I-47 lacking (−) and I-47 containing (+) mRNAs were detected by gel analysis (left) and confirmed by Sanger sequencing (right). **c** RNA from amoeba cells was analyzed by an RT-PCR approach using a primer set flanking the polyadenylation signals. A poly(T) specific primer was used in the first-strand synthesis reaction that contained a unique sequence tag at the 5′ end, corresponding to a tag-complementary downstream primer. Amplicons containing polyA tails were detected by gel analysis (left) and confirmed by Sanger sequencing (right)

To examine I-*Dsq*I expression in vivo*,* total RNA from *D. squamulosum* amoeba was isolated and subjected to RT-PCR and Sanger sequencing. We first addressed if the small spliceosomal intron (I-47) was present or not in cellular RNA (Fig. 6a). Two amplicons of expected sizes were observed, corresponding to intron lacking and intron containing RNA (Fig. 6b). Polyadenylation of I-*Dsq*I mRNA was assessed using a poly(T) specific primer with a unique sequence tag at the 5′ end in the first-strand synthesis reaction. Polyadenylated mRNA was then amplified from a primer-set consisting of an upstream primer and a downstream primer, the latter complementary to the poly(T) primer tag (Fig. 6a). The resulting amplicons were separated on an agarose gel, gel purified, and Sanger sequenced. Two polyadenylation sites were detected that correspond to the polyadenylation signal I and II located within the 3′ untranslated region of I-*Dsq*I mRNA (Fig. 6c). These experiments support that I-*Dsq*I mRNA formation involves removal of the spliceosomal intron and polyadenylation at two alternative sites.

### I-*Dsq*I is an active endonuclease that cleaves the intron-lacking rDNA locus

**Fig. 7:**
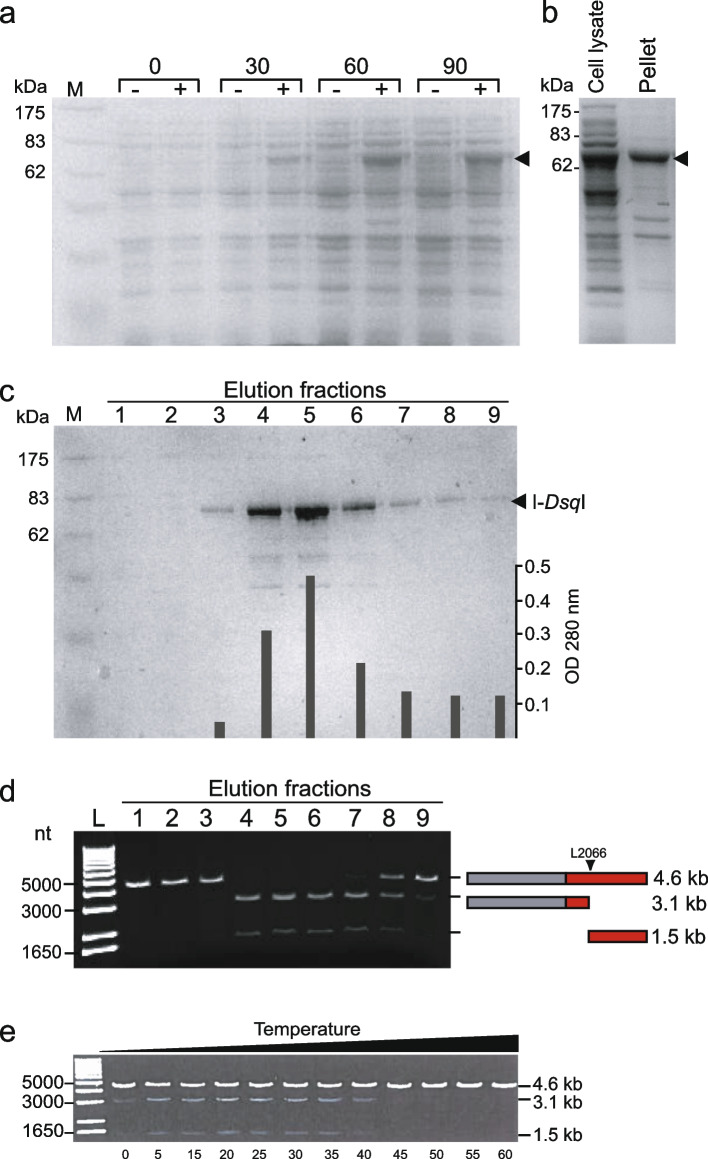
I-*Dsq*I homing endonuclease expression, purification and activity. **a** 10% SDS-PAGE gel showing expression of MBP-I-*Dsq*I in *E. coli* CodonPlus cells. Samples were harvested from induced (+) and uninduced (−) cells every thirty minutes. M is the molecular mass standard. Incubation times are shown above lanes. Arrowhead indicates the expressed endonuclease. The protein is theoretically 68 kDa. **b** After expression, the cells were lysed and centrifuged. The supernatant (cell lysate) and the pellet material (insoluble matter) were run on a 10% SDS-PAGE to clarify if MBP-I-*Dsq*I is soluble or not. Expected size of MBP-I-*Dsq*I is indicated with an arrow. **c** SDS-PAGE analysis of affinity purified MBP-I-*Dsq*I. Protein purification was executed using amylose resin. Bound MBP-I-*Dsq*I was released from the column using maltose. Nine fractions were sampled. M is a broad range prestained protein marker (New England Biolabs). Purified protein is indicated by an arrow. OD 280 nm measurements of each fraction are shown. Fraction 5 contains the highest concentration of MBP-I-*Dsq*I. **d** Activity study. The nine fractions from c) were incubated with the linarized target DNA pDan122/747 for 15 min. Two bands at 3.07 kb and 1.53 kb indicate I-*Dsq*I activity. **e** Temperature range of activity observed after incubation of 0.1 unit affinity purified I-*Dsq*I and linear target DNA for 60 min. Temperatures assessed ranged from 0 °C to 60 °C

To heterologously express the I-*Dsq*I homing endonuclease in *E. coli*, the HEG (lacking I-47) was first PCR amplified from cDNA originating from *D. squamulosum* cells, then subcloned into the pTH1 expression vector with an N-terminal *malE* fusion that encodes the maltose binding protein (MBP). MBP-I-*Dsq*I fusion protein was over-expressed after IPTG induction in *E. coli*. Figure 7a shows an SDS-PAGE gel with protein from samples harvested every 30 min after IPTG induction. A band corresponding to the size of MBP-I-*Dsq*I (approx. 68 kDa) increases in intensity in induced cells. Figure 7b shows total proteins from lysed cells, and from pelleted material after centrifugation. The presence of the expected fusion protein in both fractions indicates that the MBP-I-*Dsq*I fusion is partially soluble under the conditions used. The soluble protein phase was next subjected to affinity purification using 5 ml amylose resin. Figure 7c shows an SDS-PAGE gel with proteins from the collected fractions 1 – 9 after addition of maltose. A strong band corresponding to the size of MBP-I-*Dsq*I can be seen in fractions 4 and 5. To test for DNA endonuclease activity, aliquots of fractions 1 – 9 were incubated at 37 °C with linear target DNA containing the L2066 intron-lacking rDNA allele. After incubation and gel analysis, specific cleavages of target DNA were observed that corresponded to the expected fragment sizes, given that the enzyme cleaves the rDNA near the intron insertion site (Fig. 7d). Optimal temperature for cleavage was assessed over a broad temperature range of 0 °C to 60 °C using linear target DNA, 60 min incubation, and 0.1 unit endonuclease (see Materials & Methods). Purified I-*Dsq* I was found active over a broad range of temperatures, from 5 °C to 40 °C (Fig. 7e), which is similar to that found for the *Naegleria* I-*Nja*I homing endonuclease [23].

### I-*Dsq*I generates a pentanucleotide 3′-overhang at the L2066 intron insertion site

**Fig. 8:**
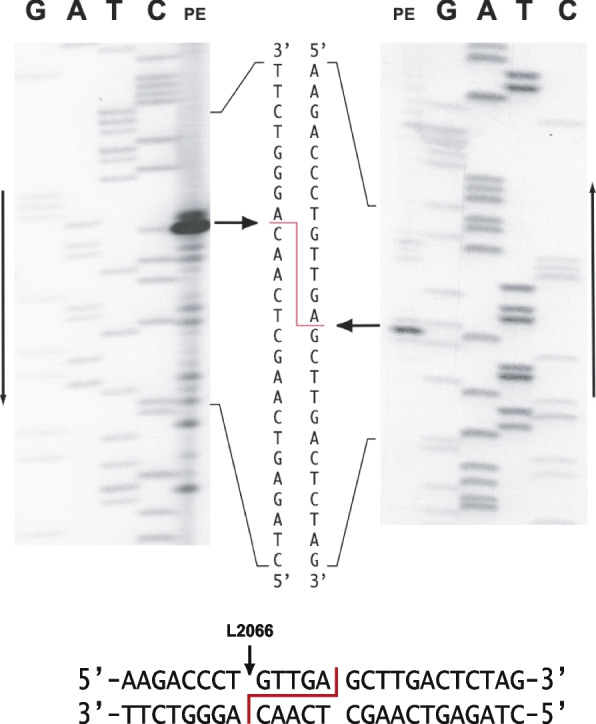
Mapping of I-*Dsq*I homing endonuclease cleavage site. The cleavage site was mapped using primer extension (PE) and Sanger sequencing in parallel when using specific primers for analysing both strands of the target DNA. Products were separated on a 5% denaturing polyacrylamide gel. Below: summary of cleavage site mapping showing a pentanucleotide 3′ overhang. Vertical arrow indicates site for intron insertion

The precise cleavage site for I-*Dsq*I was determined by primer extension analysis performed at both strands on cleaved target DNA. Sanger sequencing reactions on un-cleaved target DNA using the same primers were run in parallel. Determination of the lower strand cleavage-site (Fig. 8, left panel) indicated cleavage 3′ of a C-residue exactly at the intron insertion site. Similarly, the cleavage site of upper strand was found to be 3′ of an A-residue at position + 5 (Fig. 8, right panel). We conclude that I-*Dsq*I generates a five-nucleotide 3′ staggered cut at the L2066 intron insertion site, but with no detectable sequence symmetry (Fig. 8, lower panel).

## Discussion

We report analyses of the L2066 family of group I introns based on a dataset of 34 introns representing ascomycete and myxomycete taxa. All introns belong to the group IE subtype and possess highly conserved catalytic RNA cores. Introns from two myxomycete taxa, *D. alpinum* It-K56 and *D. squamulosum* CR10, contain large HEG insertions in the RNA segment P9. Both HEGs contain consensus polyadenylation signals in proximity to the termination codons and are interrupted by small spliceosomal introns closer to the initiation codons. Interestingly, the *D. squamulosum* HEG is located on the opposite (antisense) strand compared to its corresponding self-splicing ribozyme and host rRNA gene and appears to be expressed in myxomycete amoeba. Over-expression in *E. coli* produces an active I-*Dsq*I homing endonuclease, and primer extension analysis maps a double stranded break with a pentanucleotide 3′-overhang at the intron DNA insertion site.

HEGs in *D. alpinum* and *D. squamulosum* were found to contain spliceosomal introns of 49 bp and 47 bp, respectively. Similar spliceosomal introns have been reported in some, but not all, nuclear group I intron HEGs. These include the S943 group I intron in an Ericoid mycorrhizal fungus [37], the S1389 group I introns of Trichiales myxomycetes [32], the S516 introns of Stemonitales and Trichiales myxomycetes [33], and the S956 group I introns in two isolates of the Physarales myxomycete *D. iridis* (Fig. 4b) [24, 30, 34]. HEG spliceosomal introns may facilitate gene expression, mRNA stability, or mRNA nuclear to cytoplasmic translocation by recruiting spliceosomal components and exon junction complexes [24, 38, 39]. Furthermore, spliceosomal intron sequences may also be involved in additional processes. Recently we suggested that the I-51 spliceosomal intron in *D. iridis* interacts with the lariat capping ribozyme through strong base-pairings, and thus participate in 5′-end modification of the homing endonuclease mRNA [40].

The nuclear rDNA locus is dedicated to high-level transcription of rRNA genes, but expression of genes coding for proteins embedded in rRNA is not unprecedented. Reverse transcriptases and intron endonucleases are known to be expressed from rDNA harboring non-LTR retrotransposons and group I introns, respectively, in many Arthropoda species and eukaryotic microorganisms [25, 41]. Polyadenylation of mRNA is an indication of mRNA gene expression, and we observed consensus polyadenylation signals in proximity to HEG stop codons in both *D. alpinum* and *D. squamulosum*. Interestingly, *D. squamulosum* contains two polyadenylation signals, which are both used for polyadenylation of the mRNA in vivo. Similar polyadenylation signals, and subsequent polyadenylation, have been noted in several group I intron homing endonuclease mRNA in amoebo-flagellates [29, 31] and myxomycetes [24, 30, 32, 33].

**Fig. 9:**
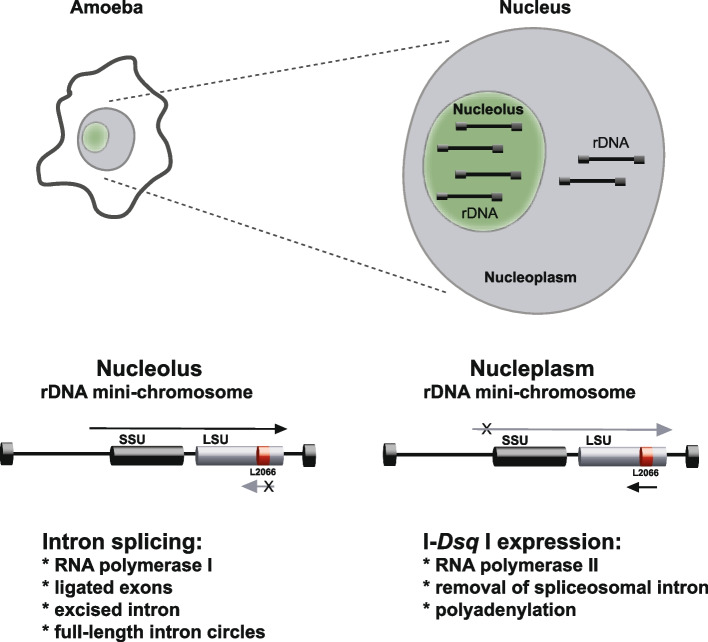
Potential model for antisense HEG expression in *Didymium* cells. Extrachromosomal rDNA copies are located both in nucleolus and nucleoplasm. The nucleolar rDNA (left) generates an RNA pol I transcript covering the SSU and LSU rRNA genes, and group I intron Dsq.L2066. Intron RNA self-splicing and processing results in ligated exons, excised intron, and full-length intron circles. The nucleoplasm rDNA (right) generates an RNA pol II transcript covering the HEG. Maturation of the corresponding I-*Dsq*I mRNA involves removal of spliceosomal intron (I-47) and polyadenylation. These two processes are separated in space and probably time, avoiding antisense interference between pre-rRNA transcripts and mRNA

Expression of the *D. squamulosum* HEG can potentially create antisense interference between the HEG mRNA and the rRNA in cells. The essential role of rRNAs in ribosomes (and thus in cell growth) can therefore represent a serious hazard for growing cells. Based on observations presented here and support by a previous study in *D. iridis* [24] we propose a potential model for spatial and probably temporal separation of transcripts that involves different promoters and polymerases (Fig. 9). 1) The rDNA in *Didymium* and other myxomycetes appear as multicopy extrachromosomal mini-chromosomes [16, 42–44]. Thus, copies of the extrachromosomal rDNA in *D. squamulosum* may be located both in the nucleolus and nucleoplasm. 2) The nucleolar rDNA will express rRNA genes from a regular RNA pol I promoter, and subsequently the L2066 intron become excised during ribozyme-catalyzed RNA processing. 3) The nucleoplasm rDNA will express the I-*Dsq*I HEG from a corresponding RNA pol II promoter located on the opposite strand, generating an I-47 lacking and polyadenylated mRNA. 4) Since the RNA pol I and RNA pol II transcriptions are mainly restricted to the nucleolus and nucleoplasm, respectively, in eukaryotic cells, antisense interference between the pre-rRNA and I-*Dsq*I mRNA becomes minimized or avoided.

Homing of a nuclear rDNA group I intron has been reported in experimental settings in two myxomycete species, *P. polycephalum* and *D. iridis* [15, 16]. Homing is dependent on sexual mating and is initiated by a DNA double strand break at the intron insertion site in an intron-lacking allele, catalyzed by the intron-encoded endonuclease. I-*Dsq*I contains strong hallmarks of a His-Cys homing endonucleases, including conserved amino acid residues in zinc coordination and catalysis, and the ability to cleave an intron-lacking allele DNA at the insertion site. I-*Dsq*I was found to generate a pentanucleotide 3′-overhang at the cleavage site. This was similar to that of *Naegleria* I-*Nja*I, I-*Nit*I, and I-*Nan*I homing endonucleases [22, 23] and *Didymium* I-*Dir*II [24], but different from the tetranucleotide 3′-overhang made by I-*Ppo*I from *Physarum* [20, 21].

## Conclusions

Thirty-four nuclear group IE introns at position L2066 from ascomycete and myxomycete rDNAs were analyzed, and mobile-type introns were found in two of the myxomycete taxa; *D. alpinum* It-K56 and *D. squamulosum* CR10. Both introns harbor His-Cys HEGs interrupted by spliceosomal introns*.* In vivo expression of the antisense-organized HEG in *D. squamulosum* was supported by mRNA polyadenylation and the removal of a 47-nt spliceosomal intron. The corresponding endonuclease protein, I-*Dsq*I, was over-expressed in *E. coli* and showed DNA cleavage activity corresponding to a pentanucleotide 3′ overhang at the intron-lacking allele. These features are consistent with group I intron mobility. Finally, we propose a potential model to explain how an antisense HEG from a mobile group I intron become expressed from a nuclear rDNA locus due to spatial and probably temporal separation of rRNA and mRNA transcriptions.

## Materials and methods

### Myxomycete strain, nucleic acid isolation, amplifications, and rDNA sequencing

*D. squamulosum* was generously provided by Dr. Jim Clark (University of Kentucky). Approximately 10^8^ amoeba cells were harvested from DS/2 agar plates, and total DNA extraction and rDNA sequencing were performed as described previously [45]. Extraction of total cellular RNA, PCR, RT-PCR and Sanger sequencing were performed as described previously [24, 29, 46]. Primer information: OP1019, 5′-TCA GAG CGT TGG CTG TTC-3′; OP1020, 5′-AAT CTT GAG TTC GTG GTA GC-3′; OP1021, 5′-ACG TCT ACT TTG CGA GC-3′, OP313, 5′-AAG CG ACGC ATG CACGCA TT-3′; OP41, 5′-CGA CGC ATG CAC GCA TTT TTT TTT TTT TTT-3′.

### Phylogenetic analysis

The tree-building methods of Neighbor joining (NJ) and Minimum Evolution (ME) interpreted in Geneious prime® 2019.2.3. and MEGA X [47] with default settings. The evolutionary history of intron dataset 1 was reconstructed with NJ and the Jukes-Cantor (JC) model using MEGA X with default settings. The robustness of the nodes of the tree was tested with NJ-JC (500 replicates) and ME-JC (500 replicates).

### In vitro transcription and group I intron splicing

Dsq.L2066 with some flanking exon sequences (approximately 50 nt of the 5′ exon and 150 nt of the 3′ exon), were PCR amplified from LSU rDNA and inserted into pGEM-T easy vector (Promega) downstream the T7 promoter. Intron containing RNAs were transcribed from linearized plasmid using T7 RNA polymerase (Stratagene, La Jolla, CA, USA) and RNA species of interest were eluted after PAGE separation and submitted to RT-PCR Sanger sequencing as described previously [29, 48]. Primer information: OP808, 5′-AAT TTA ATA CGA CTC ACT ATA GGG CTT GGC ACA ATT AGC GG-3′; OP809, 5′-GTT AGT TAC AGC ATT AGC-3′.

### Plasmid construction

The I-*Dsq*I HEG, containing or lacking the I-47, was introduced into expression vector pTH1 (Addgene). pTH1 generates N-terminal maltose binding protein (MBP) fusions. Briefly, the I-*Dsq*I HEG was PCR amplified using cDNA made from total RNA from *D. squamulosum* and the first-strand oligonucleotide primer OP41, using the primer pair OP1422 and OP1423. The resulting entry clone pDONR221-I-*Dsq*I was used to introduce the HEG into the pTH1 expression vector in an LR reaction. Final construct for N-terminal MBP fusion was named pI-*Dsq*I-MBP. Target DNA for homing endonuclease cleavage was made by insertion of a 1.59 kb fragment of a L2066-lacking *Didymium* LSU rDNA (amplified by OP122 and OP747) into the pGEM-T easy vector. The plasmid was linearized with *Nde*I restriction enzyme (New England Biolabs) prior to activity studies. Primer information: OP122, 5′-CGC GCA TGA ATG GAT TA-3′; OP747, 5′-TCC AAC ACT TAC TGA ATT CT-3′; OP1422, 5′-GGG GAC AAG TTT GTA CAA AAA AGC AGG CTT CAT GAA CAA CTA CCA GCA GGC A-3′; OP1423, 5′-GGG GAC CAC TTT GTA CAA GAA AGC TGG GTC CTA CCA GCA TGC TGG GGT GTG GTT-3′.

### Protein expression

For expression of fusion proteins pI-*Dsq*I-MBP was introduced into *E. coli* BL21-CodonPlus (DE3)-RIL cells (Stratagene). Cells were cultured in YT-medium with 100 μg/ml ampicillin and 100 μg/ml chloramphenicol. Cultures were grown to OD_600 nm_ 0.4–0.6, then split into two halves, and expression of fusion proteins was induced in one half by adding Isopropyl-1-thio-β-D-galactopyranoside (IPTG) or 20% L-arabinose, respectively. The one half of the culture with no added arabinose was grown in parallel and used as negative control. Samples of 0.5 ml were harvested every 30 min and subjected to SDS-PAGE to monitor expression of proteins. 90 min after induction the cells were harvested by centrifugation for 20 min at 4000 g (5000 rpm in a Sorwall GSA rotor, Dupont Instruments). The cells were resuspended in 5 ml column buffer and stored over night at − 20 °C. Next day samples were thawed in ice-cold water before they were sonicated in six short pulses of 10 sec with 20-sec pause while kept on ice. After centrifugation for 20 min at 4 °C at 14000 g (11,000 rpm in a Sorwall SS-34 rotor, Dupont Instruments) the supernatant (crude extract) was stored on ice. The pellet was resuspended in column buffer (insoluble matter). Samples of supernatant and resuspended pellet were subjected to SDS-PAGE to monitor solubility of fusion proteins.

### Affinity purification of proteins

I-*Dsq*I-MBP fusion proteins were affinity purified by running crude cell extract through a 16 × 100 mm column packed with 5 ml amylose resin, with the flow rate set at 0.5 ml/min. The column was subsequently washed with 5 column volumes of distilled water and 10 column volumes column buffer. I-*Dsq*I-MBP was eluted by before 5 ml crude extract was added. To release the fusion protein from the matrix column buffer containing 10 mM maltose was added to the column and 1.5 ml fractions were collected. Samples from each fraction were subjected to 10% SDS-PAGE and OD_280 nm_ measurements to monitor protein purification.

### In vitro homing endonuclease cleavage assays and cleavage site mapping

I-*Dsq*I endonuclease activity was assessed by incubating 1 μg linearized target DNA in a 20 μl reaction containing 10 x Tris Acetate-EDTA (TAE) buffer (1 x TAE buffer with 40 mM Tris-acetate and 1 mM EDTA at pH 8.3), 0.1 unit of enzyme and 60 min incubation at 37 °C, and separated on 0.7% agarose gels after incubation. One unit of I-*Dsq*I was defined as the amount of enzyme required to completely digest 1 μg linearized target DNA in 1 h at 37 °C in a 20 μl reaction containing 10 x TAE buffer. Cleavage site mapping was performed essentially as described previously [22, 23] based on primer extension analyses on I-*Dsq*I cleaved linearized target DNA using OP122 (located ca 105 nt upstream cleavage site) and OP1451 (located ca 87 nt downstream cleavage site) and α-^33^P dATP as the label. Primer extension products were separated on a 5% polyacrylamide gel alongside manual Sanger sequencing reactions generated from un-cleaved target DNA using the same primers. Primer information: OP1451, 5′-GGT GGT ATT TCA AGG TCG-3′.

## Supplementary Information

**Additional file 1: Figure S1.** Consensus secondary structure diagrams of L2066 group I introns in myxomycetes and ascomycetes. **a**) Consensus structure in myxomycetes based on ca 250 nucleotide positions in the catalytic core common among introns from 16 taxa (see Table 1). Sequence size variations are noted in most peripheral regions, and homing endonuclease genes (HEGs) are found as P9 extensions. P1-P10 and P13, paired RNA segments; 5′ SS and 3′SS, exon-intron splice sites. Invariant nucleotide positions are shown as red uppercase letters. Black uppercase letter, > 90% conservation; lowercase letters, ≥ 50% conservation; filled circles, < 50% conservation. **b**) Consensus structure in ascomycetes based on ca 260 nucleotide positions in the catalytic core common among introns from 18 taxa (see Table 1).

**Additional file 2: Figure S2.** Sequence alignment of 186 core structure nucleotides of myxomycete and ascomycete L2066 group I introns. Secondary structure paired segments (P1-P8) are shown above the alignment, and key segments are colour coded. Intron taxa sequences are indicated by species name abbreviations and GeneBank accession numbers.

## Data Availability

Sequencing data are available in GenBank under the accession numbers ON155995 (*Lepidoderma alpestroides*), ON155996 (*Comatricha laxa*), and ON155997 (*Fusarium* sp.).

## Abbreviations

exoG: Exogenous guanosine cofactor
FLC: Full length circularization;
HEG: Homing endonuclease gene
LSU rRNA: Large subunit ribosomal RNA
rDNA: Ribosomal DNA
SS: Splice site
SSU rRNA: Small subunit ribosomal RNA
