# Supplementary figures and images for: Mobile group I introns at nuclear rDNA position L2066 harbor sense and antisense homing endonuclease genes intervened by spliceosomal introns
Source: Mob DNA. 2022 Oct 8;13:23. doi: 10.1186/s13100-022-00280-4 (PMC9548176; doi:10.1186/s13100-022-00280-4)

a

Myxomycete L2066 consensus

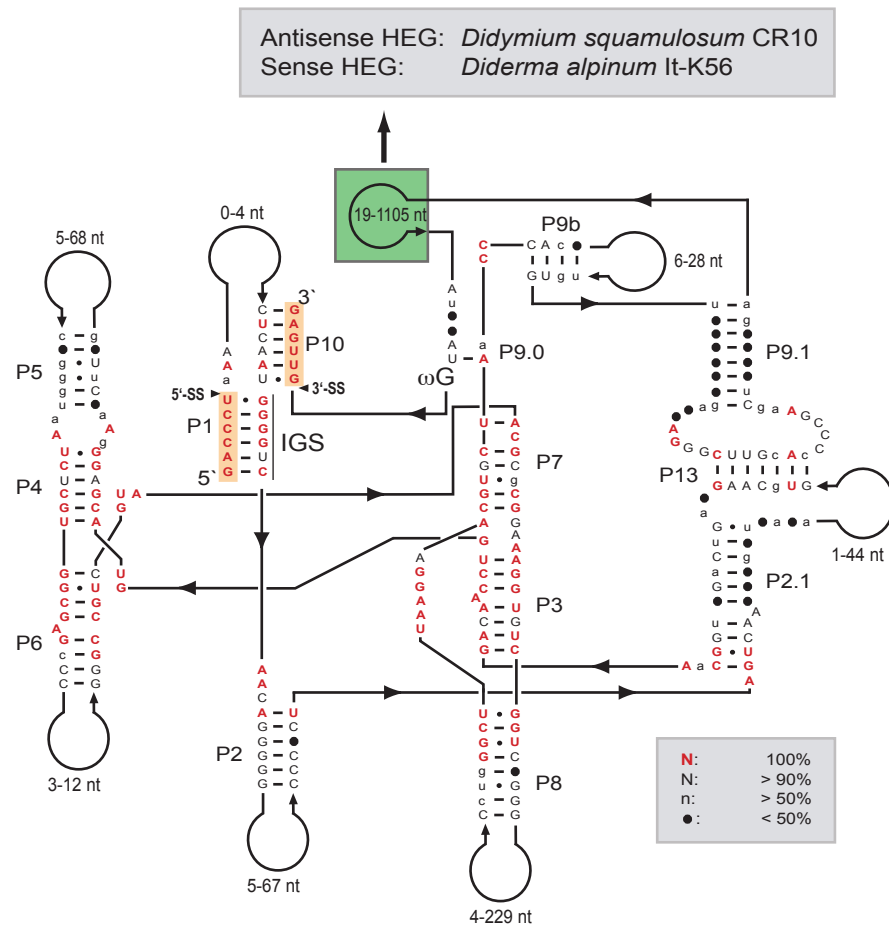

b

Ascomycete L2066 consensus

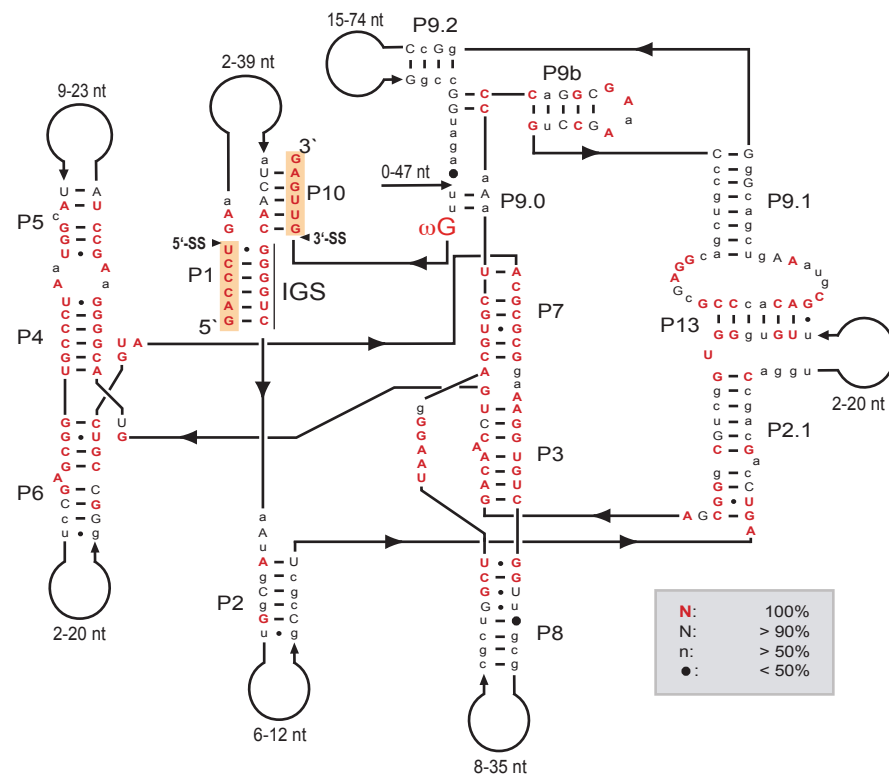

Figure S1

Supplement: Supplementary file 1 — Additional file 1: Figure S1. Consensus secondary structure diagrams of L2066 group I introns in myxomycetes and ascomycetes. a) Consensus structure in myxomycetes based on ca 250 nucleotide positions in the catalytic core common among introns from 16 taxa (see Table 1). Sequence size variations are noted in most peripheral regions, and homing endonuclease genes (HEGs) are found as P9 extensions. P1-P10 and P13, paired RNA segments; 5′ SS and 3′SS, exon-intron splice sites. Invariant nucleotide positions are shown as red uppercase letters. Black uppercase letter, > 90% conservation; lowercase letters, ≥ 50% conservation; filled circles, < 50% conservation. b) Consensus structure in ascomycetes based on ca 260 nucleotide positions in the catalytic core common among introns from 18 taxa (see Table 1). [file 13100_2022_280_MOESM1_ESM.pdf]
