# Supplementary material for: Mobile group I introns at nuclear rDNA position L2066 harbor sense and antisense homing endonuclease genes intervened by spliceosomal introns
Source: Mob DNA. 2022 Oct 8;13:23. doi: 10.1186/s13100-022-00280-4 (PMC9548176; doi:10.1186/s13100-022-00280-4)
Supplement: Supplementary file 2 — Additional file 2: Figure S2. Sequence alignment of 186 core structure nucleotides of myxomycete and ascomycete L2066 group I introns. Secondary structure paired segments (P1-P8) are shown above the alignment, and key segments are colour coded. Intron taxa sequences are indicated by species name abbreviations and GeneBank accession numbers. [file 13100_2022_280_MOESM2_ESM.pdf]

Figure S2: L2066_Consencus alingment (34 introns)

```
              <<P1>>      <<P10><<P1>>    <<<<<<P2>>>>>> <P2.1><<P13>><P2.1>  <<<P3>>>
Cmi_HE655081  gaccctAAA//TTCGATGGGGTCAACAGGGGA//TCCTCTAGTCAA/GTGCAAG/TTGGCGAGACAACCTGGT
Dal_HE655057  gaccctAAA//CTCAATGGGGTCAACAGGGGG//CCCTCTAGTCAC/GTGCAAG/TTGGCAAGACAACCTGGT
Dal_HE655058  gaccctAAA//CTCAATGGGGTCAACAGGGGG//CCCTCTAGTCAC/GTGCAAG/TTGGCAAGACAACCTGGT
Dme_HE655059  gaccctGAA//CTCAATGGGGTCAACAGGGGG//CCTCCTAGTCAA/GTGCAAG/CTGGCAAGACAACCTGGT
Dni_AM407429  gaccctGAA//CTCAACGGGGTCAACAGGGGG//CCCTCTAGTCAA/GTACAAG/GTGGCAAGACAACCTGGT
Dni_HE655060  gaccctGAA//CTCAACGGGGTCAACAGGGGG//CCCTCTAGTCAA/GTACAAG/GTGGCAAGACAACCTGGT
Dni_HE655061  gaccctGAA//CTCAACGGGGTCAACAGGGGG//CCCTCTAGTCAA/GTACAAG/GTGGCAAGACAACCTGGT
Dso_AM407428  gaccctAAA//CTCAATGGGGCCAACAGGGGG//CCTCCTAGTCGT/GTACAAG/GCGGCGAGACAACCTGGT
Dsq_AM407427  gaccctGAA//CTCAATGGGGTCAACAGGGGG//CCCTCTAGTTGA/CTGTGAG/CCAGCAAGACAACCTGGT
Fse_HE655082  gaccctAAA//CTCAATGGGGCCAACAGGGGG//CCCCCTAGTCGA/GTGCAAG/CCGGCGAGACAACCTGGT
Lag_HE655062  gaccctAAA//CTCAATGGGGTCAACAGGGGG//CCCCCTAGTCAA/ATGCAAG/CTGGCGAGACAACCTGGT
Lal_ON155995  gaccctAAA//CTCAATGGGGTCAACAGGGGG//CCCCCTAGTCAA/GTGCAAG/TTGGCAAGACAACCTGGT
Lca_AM407430  gaccctAAA//CTCAATGGGGTCAACAGGGGG//CCCCCTAGTCAA/ATGCAAG/CTGGCGAGACAACCTGGT
Lcr_HE655064  gaccctAAA//CTCAATGGGGCCAACAGGGGG//CCTCCTAGTCAC/GTACAAG/CTGGCAAGATAACCTGGT
Mcr_HE655067  gaccctAAA//CTCAATGGGGTCAACAGGAGG//CCTTCTAGTTGA/GTGCAAG/TCAGCAAGACAACCTGGT
Cla_ON155996  gaccctAAG//TTGCATGGGGTCAAAAGGGCG//AGCCCTAGTCAA/GTGCAAG/CTGGCGAGACAACCTGGT
Squ_GU214496  gaccctGAA//CTCAACGGGGTCAAAAGTAGT//GCTGCTAGTCTC/TTGCAGG/CAGGCCAGACAACGTGGT
Bba_EU334679  gaccctGAA//ATCAACGGGGTCAATAGCGGT//GCCGCTAGTCCA/TTGTGGG/CGGGCGAGACAACCTGGT
Bba_KJ701419  gaccctGAA//ATCAACGGGGTCAATAGCGGT//GCCGCTAGTCCA/TTGTGGG/CGGGCGAGACAACCTGGT
Bba_KJ701420  gaccctGAA//ATCAACGGGGTCAATAGCGGT//GCCGCTAGTCCA/TTGTGGG/CGGGCGAGACAACCTGGT
Bba_EU334676  gaccctGAA//ATCAACGGGGTCAATAGCGGT//GCCGCTAGTCCA/TTGTGGG/CGGGCGAGACAACCTGGT
Bba_MG654725  gaccctGAA//ATCAACGGGGTCAATAGCGGT//GCCGCTAGTCCA/TTGTGGG/CGGGCGAGACAACCTGGT
Bba_MG654726  gaccctGAA//ATCAACGGGGTCAATAGCGGT//GCCGCTAGTCCA/TTGTGGG/CGGGCGAGACAACCTGGT
Bba_JF429894  gaccctGAA//ATCAACGGGGTCAATAGCGGT//GCCGCTAGTCCA/TTGTGGG/CGGGCGAGACAACCTGGT
Cka_AB044639  gaccctGAA//CTCAACGGGGTCGAAAACGGC//GCCGTTAGTCCA/TTGCAGG/CGGGCGAGACAACCTGGT
Cmi_CP023322  gaccctGAA//ATCAACGGGGTCAATAGCGGT//GCCGCTAGTCCA/TTGTGGG/CGGGCGAGACAACCTGGT
Cpr_AB044641  gaccctGAA//ATCAACGGGGTCAGTAGCGCC//GCCGCTAGTCCA/CTGTGGG/CGGGCGAGACAACCTGGT
Fsp_ON155997  gaccctGAG//CTCAACGGGGTCAACAATAGC//GCTATTAGTCCA/TTACGGG/CGGGCGAGACAACCTGGT
Lsp_MH013330  gaccctGAA//ATCAACGGGGTCAAGAGCGGT//GCCGCTAGTCCA/TTGTGGG/CGGGCGAGACAACCTGGT
Osi_FJ461354  gaccctGAG//CTCAACGGGGTCGAGAACGGC//GCCGTTAGTCCA/CTGCAGG/CGGGCGAGACAACCTGGT
Osi_FJ461355  gaccctGAG//CTCAACGGGGTCGAGAACGGC//GCCGTTAGTCCA/CTGCAGG/CGGGCGAGACAACCTGGT
Pte_AB044642  gaccctGAA//ATCGACGGGGTCAAGAGCGGT//AGCCTCAGTCCG/CTGTGGG/CGGGCGAGACAACCTGGA
Mmo_DQ518989  gaccctGAA//CATAACGGGGTCAAAAGCAGT//GCTGCTAGTCTA/TTGTGGG/CGGGCGAGACAACCTGGT
Mni_DQ518993  gaccctGAA//ATCAACGGGGTCAATAGCAGT//ACTGCTAGTCTA/TTGTGGG/CAGGCGAGACAACCTGGA
              <<P1>>      <<P10><<P1>>    <<<<<<P2>>>>>> <P2.1><<P13>><P2.1>  <<<P3>>>
```

```
              <<P4>>  <<<P5>>>  <<P4>><<<<<<<<<P6>>>>>>>>    <<<P7>>    <<P3>>><<<<<<<<P8
Cmi_HE655081  ACGAGGGAGGC//TGCTATCTCGTGGCGAGCCT//GGGCCGTCGTAACGCGCGGAAAGGTGTCGGTCGGAG//
Dal_HE655057  ACGAGGAAAAT//GACAATCTCGTGGCGAGCCC//AGGCCGTCGTAACGTGCGGAAAGGTGTCGGTCTAGA//
Dal_HE655058  ACGAGGAAAAT//GACAATCTCGTGGCGAGCCC//AGGCCGTCGTAACGTGCGGAAAGGTGTCGGTCTAGA//
Dme_HE655059  ACGAGGAAAAC//GACAATCTCGTGGCGAGCCC//AGGCCGTCGTAACGCGCGGAAAGGTGTCGGTCCGGG//
Dni_AM407429  ACGAGGAAGGC//AGTAATCTCGTGGCGAGTCC//GGGCCGTCGTAACGCACGCAAAGGTGTCGGTCCGGG//
Dni_HE655060  ACGAGGAAGGC//AGTAATCTCGTGGCGAGTCC//GGGCCGTCGTAACGCACGCAAAGGTGTCGGTCCGGG//
Dni_HE655061  ACGAGGAAGGC//AGTAATCTCGTGGCGAGTCC//GGGCCGTCGTAACGCACGCAAAGGTGTCGGTCCGGG//
Dso_AM407428  ACGAGGGAAAC//GGTAATCTCGTGGCGAGTCG//TGGCCGTCGTAACGCACGGTAAGGTGTCGGTCGGGG//
Dsq_AM407427  ACGAGGGAAAC//GATAATCTCGTGGCGAGCCC//AGGCCGTCGTAACGCGCGGTAAGGTGTCGGTTAGGG//
Fse_HE655082  ACGGGGGAGCC//GGGAATCTCGTGGCGAGCTA//AGGCCGTTGTAACGCACGGAAAGGTGTCGGTCCGTA//
Lag_HE655062  ACGAGGGAAGC//GGTCATCTCGTGGCGAGCCC//GGGCCGTCGTAACGCGCGGAAAGGTGTCGGTCTGGG//
Lal_ON155995  ACGAGGGAAGC//GGTCATCTCGTGGCGAGTCC//GGGCCGTCGTAACGCGCGGAAAGGTGTCGGTTTGGG//
Lca_AM407430  ACGAGGGAAGC//GGTCATCTCGTGGCGAGCCC//GGGCCGTCGTAACGCGCGGAAAGGTGTCGGTCTGGG//
Lcr_HE655064  ACGAGGAAGGC//AGTGATCTCGTGGCGAGCCC//GGGCCGTCGTAACGCACGGTAAGGTATCGGTTCGGG//
Mcr_HE655067  ACGAGGGAACT//GACGATCTCGTGGCGAGCTC//GAGCCGTCGTAACGTGCGGAAAGGTGTCGGTCTAGG//
Cla_ON155996  ACGAGGGAAAG//GCCTATCTCGTGGCGAGCCT//GGGCCGTCGTAACGCGCGCAAAGGTGTCGGTCTAGG//
Squ_GU214496  ACGGGGAAGCC//GGTAATCCCGTGGCGAGCCC//GGGCCGTCGTAACGCGCGGTAAGGTGTCGGCTGGGT//
Bba_EU334679  ACGGGGAAGCC//GGTGATCCCGTGGCGAGCCT//GGGCCGTCGTAACGCGCGGAAAGGTGTCGGTTAGCG//
Bba_KJ701419  ACGGGGAAGCC//GGTGATCCCGTGGCGAGCCT//GGGCCGTCGTAACGCGCGGAAAGGTGTCGGTTAGCG//
Bba_KJ701420  ACGGGGAAGCC//GGTGATCCCGTGGCGAGCCT//GGGCCGTCGTAACGCGCGGAAAGGTGTCGGTTAGCG//
Bba_EU334676  ACGGGGAAGCC//GGTGATCCCGTGGCGAGCCT//GGGCCGTCGTAACGCGCGGAAAGGTGTCGGTTAGCG//
Bba_MG654725  ACGGGGAAGCC//GGTGATCCCGTGGCGAGCCT//GGGCCGTCGTAACGCGCGGAAAGGTGTCGGTCAGCG//
Bba_MG654726  ACGGGGAAGCC//GGTGATCCCGTGGCGAGCCT//GGGCCGTCGTAACGCGCGGAAAGGTGTCGGTCAGCG//
Bba_JF429894  ACGGGGAAGCC//GGTGATCCCGTGGCGAGCCT//GGGCCGTCGTAACGCGCGGAAAGGTGTCGGTCAGCG//
Cka_AB044639  ACGGGGAAGCC//GGTAATCCCGTGGCGAGCCC//GGGCCGTCGTAACGCGCGGTAAGGTGTCGGTCGTTG//
Cmi_CP023322  ACGGGGGAGCC//GGTAATCCCGTGGCGAGCCT//GGGCCGTCGTAACGCGCGGAAAGGTGTCGGTTGGCG//
Cpr_AB044641  ACGGGGGAGCC//GGTAATCCCGTGGCGACCTT//GAGGCGTCGTAACGCGCGGAAAGGTGTCGGTTGGCG//
Fsp_ON155997  ACGGGGGAGCC//TGGAATCCCGTGGCGAGCTC//GAGCCGTCGTAACGCGCGGTAAGGTGTCGGTCTCGG//
Lsp_MH013330  ACGGGGAAGCC//GGTAATCCCGTGGCGAGCCT//GGGCCGTCGTAACGCGCGGAAAGGTGTCGGTTGGCG//
Osi_FJ461354  ACGGGGGAGCC//GGTAATCCCGTGGCGAGCCC//GGGCCGTCGTAACGCGCGGAAAGGTGTCGGTCCCCG//
Osi_FJ461355  ACGGGGGAGCC//GGTAATCCCGTGGCGAGCCC//GGGCCGTCGTAACGCGCGGAAAGGTGTCGGTCCCCG//
Pte_AB044642  ACGGGGGAGCC//GGTAATCCCGTGGCGAGTCC//GGGCCGTCGTAACGCGCGCCAAGGTGTCGGTTGGTG//
Mmo_DQ518989  ACGGGGGAGCC//GGTAATCCCGTGGCGAGCTG//TGGCCGTCGTAACGCGCGCTAAGGTGTCGGTTGGGG//
Mni_DQ518993  ACGGGGAAGCC//GGTAATCCCGTGGCGAGCTG//AGGCCGTCGTAACGCGCGGAAAGGTGTCGGTTGAGT//
              <<P4>>  <<<P5>>>  <<P4>><<<<<<<<<P6>>>>>>>>    <<<P7>>    <<P3>>><<<<<<<<P8
```

```
                >>>>>>>>          <<P7>>    <<<<P9b>>>      <<P13>>           <P10>>
Cmi_HE655081    CTCTGGCTTAAGGAACGTGCTAA//CCAC//GTGGCTGG//CTTGCAC//CCGG//TTGgttgag
Dal_HE655057    TCTAGGCTTAAGGAACGTACTAA//CACA//TGTGCCTA//CTTGCAT//TAGG//ATGgttgag
Dal_HE655058    TCTAGGCTTAAGGAACGTACTAA//CACA//TGTGCCTA//CTTGCAT//TAGG//ATGgttgag
Dme_HE655059    CCCGGGCTTAAGGAACGTACTAA//CACA//TGTGCCTG//CTTGCAT//TAGA//ATGgttgag
Dni_AM407429    CTCGGGCTTAAGGAACGTGCTAT//CACG//TGTGTTCT//CTTGTAC//AGAA//AAAgttgag
Dni_HE655060    CTTGGGCTTAAGGAACGTGCTAT//CACG//TGTGTTCT//CTTGTAC//AGAA//AAAgttgag
Dni_HE655061    CTTGGGCTTAAGGAACGTGCTAT//CACG//TGTGTTCT//CTTGTAC//AGAA//AAAgttgag
Dso_AM407428    CCCCGGCTTAAGGAACGTGCTAA//CACA//TGTGCCTG//CTTGTAC//CAGG//CTGgttgag
Dsq_AM407427    CCTTGGCTTAAGGAACGTGCTAT//CCTG//CAGGTGCT//CTCACAG//ACAA//ATGgttgag
Fse_HE655082    AACGGGCTTAAGAAACGTGCTAA//TACA//TGTACTGG//CTTGCAC//CTGG//TTGgttgag
Lag_HE655062    CCTGGGCTTAAGGAACGTGCTAA//CACA//TGTGTCTG//CTTGCAT//TGGA//ATGgttgag
Lal_ON155995    CCTGGGCTTAAGGAACGTGCTAA//CACG//CGTGTTCA//CTTACAC//TGAG//TTGgttgag
Lca_AM407430    CCTGGGCTTAAGGAACGTGCTAA//CACA//TGTGTCTG//CTTGCAT//TGGA//ATGgttgag
Lcr_HE655064    CCTGGGCTTAAGGAACGTGCTAT//CACA//TGTGTTCC//CTTGTAC//GGAA//AAGgttgag
Mcr_HE655067    CCTAGGCTTAAGGAACGTACTAA//CATG//CATGCCGG//CTTGCAC//CCGA//TTGgttgag
Cla_ON155996    CCTAGGCTTAAGGAACGTGCTAT//CGTG//CACGCCTG//CTTGCAC//CCGA//GAGgttgag
Squ_GU214496    GCCTGGCTTAAGGGACGTGCTAA//CATG//CATGTTCT//CCTGCAG//AGGA//TTGgttgag
Bba_EU334679    CGCTGGCTTAAGGGACGTGCTAA//CAGG//CCTGCCTG//CCCACAG//CGGG//TCGgttgag
Bba_KJ701419    CGCTGGCTTAAGGGACGTGCTAA//CAGG//CCTGCCTG//CCCACAG//CGGG//TCGgttgag
Bba_KJ701420    CGCTGGCTTAAGGGACGTGCTAA//CAGG//CCTGCCTG//CCCACAG//CGGG//TCGgttgag
Bba_EU334676    CGCTGGCTTAAGGGACGTGCTAA//CAGG//CCTGCCTG//CCCACAG//CGGG//TCGgttgag
Bba_MG654725    CGCTGGCTTAAGGGACGTGCTAA//CAGG//CCTGCCTG//CCCACAG//CGGG//TCGgttgag
Bba_MG654726    CGCTGGCTTAAGGGACGTGCTAA//CAGG//CCTGCCTG//CCCACAG//CGGG//TCGgttgag
Bba_JF429894    CGCTGGCTTAAGGGACGTGCTAA//CAGG//CCTGCCTG//CCCACAG//CGGG//TCGgttgag
Cka_AB044639    TCTCGGCTTAAGGTACGTGCTAA//CAGG//CCTGCGCC//CCTGCAG//GGCG//ATGgttgag
Cmi_CP023322    CGCTGGCTTAAGGGACGTGCTAA//CAGG//CCTGCCCG//CCCACAG//CGGG//ATGgttgag
Cpr_AB044641    CGCCGGCTTAAGGGACGTGCTAA//CTGG//CCAGCCCG//CCCCCAG//CGGG//TTGgttgag
Fsp_ON155997    CCGGGGCTTAAGGTACGTGCTAA//CAGG//CCTGTGCC//CCCGTAG//GGCA//TTGgttgag
Lsp_MH013330    CGCTGGCTTAAGGGACGTGCTAA//CAGG//CCTGCCCG//CCCACAG//CGGG//ATGgttgag
Osi_FJ461354    CCGGGGCTTAAGGTACGTGCTGA//CGGG//CCCGCGCC//CCTGCAG//GGCG//TTGgttgag
Osi_FJ461355    CCGGGGCTTAAGGTACGTGCTGA//CGGG//CCCGCGCC//CCTGCAG//GGCG//TTGgttgag
Pte_AB044642    CGCCGGCTTAAGGTACGTGCTAA//CAGG//CCTGCCCG//CCCACAG//CGGG//ATGgttgag
Mmo_DQ518989    CCCCGGCTTAAGGTACGTGCTAA//CTGG//CCTGCCCT//CCCACAG//AGGG//ATGgttgag
Mni_DQ518993    AATCAGCTTAAGGGACGTGCTAT//CAGG//CCTGCTGT//CCCACAG//ACGG//ATGgttgag
                >>>>>>>>          <<P7>>    <<<<P9b>>>      <<P13>>           <P10>>
```
